# Regulated Hyaluronan Synthesis by Vascular Cells

**DOI:** 10.1155/2015/208303

**Published:** 2015-09-10

**Authors:** Manuela Viola, Evgenia Karousou, Maria Luisa D'Angelo, Ilaria Caon, Giancarlo De Luca, Alberto Passi, Davide Vigetti

**Affiliations:** Department of Surgical and Morphological Sciences, University of Insubria, 21100 Varese, Italy

## Abstract

Cellular microenvironment plays a critical role in several pathologies including atherosclerosis. Hyaluronan (HA) content often reflects the progression of this disease in promoting vessel thickening and cell migration. HA synthesis is regulated by several factors, including the phosphorylation of HA synthase 2 (HAS2) and other covalent modifications including ubiquitination and O-GlcNAcylation. Substrate availability is important in HA synthesis control. Specific drugs reducing the UDP precursors are able to reduce HA synthesis whereas the hexosamine biosynthetic pathway (HBP) increases the concentration of HA precursor UDP-N-acetylglucosamine (UDP-GlcNAc) leading to an increase of HA synthesis. The flux through the HBP in the regulation of HA biosynthesis in human aortic vascular smooth muscle cells (VSMCs) was reported as a critical aspect. In fact, inhibiting O-GlcNAcylation reduced HA production whereas increased O-GlcNAcylation augmented HA secretion. Additionally, O-GlcNAcylation regulates HAS2 gene expression resulting in accumulation of its mRNA after induction of O-GlcNAcylation with glucosamine treatments. The oxidized LDLs, the most common molecules related to atherosclerosis outcome and progression, are also able to induce a strong HA synthesis when they are in contact with vascular cells. In this review, we present recent described mechanisms involved in HA synthesis regulation and their role in atherosclerosis outcome and development.

## 1. Introduction

Cardiovascular diseases, which include heart attacks, strokes, and peripheral vascular disease, are the first cause of death in Asia, Europe, and United States [1]. Atherosclerosis is the underlying cause of such vascular disorders, and even though its pathogenesis has been actively investigated in the last decades, several aspects are still elusive. Among the factors involved in plaque formation and progression as well as in vessel thickening (i.e., neointima formation after vascular surgery such as angioplasty or stent placement), extracellular matrix (ECM) plays a pivotal role [2]. ECM composition modifies the behavior of different cell types, including the vascular cells. In fact, the cell microenvironment is involved in most diseases by altering several cell functions. Furthermore, several factors influence the ECM architecture promoting endothelial dysfunction [3, 4].

Arteries consist of three layers, known also as tunica, named intima, media, and adventitia, whose architecture differs in cell population and ECM composition. The intima layer, which is in contact with the bloodstream, is mainly composed of endothelial cells (ECs) attached to the basement membrane. The media are composed of smooth muscle cells (SMCs) producing elastin and small amounts of ECM. The adventitia, the outermost layer, is made by a sheath of fibroblasts with a dense ECM.

Atherosclerotic lesions consist of asymmetric intima thickenings due to a chronic inflammatory response of the arterial wall, initiated by injury caused by hyperglycemia, modified low-density lipoprotein (LDL), or hypertension [5]. The earliest changes that lead to formation of atherosclerotic lesions are due to EC dysfunction [3] and include lowered production of nitric oxide or augmented lipoprotein permeability, immune cell adhesion (by increased ICAM and VCAM), and thrombotic potential [6]. The recruitment of monocytes, which differentiate into macrophages in the intima, is the signal for the secretion of proinflammatory chemokines, including IL-1beta and TNF alpha. Moreover, activated macrophages produce reactive oxygen species that cause LDL oxidation. In fact, when LDL particles become trapped in the vessel wall, they undergo progressive oxidation, and they can be internalized by macrophages through scavenger receptors, which are not regulated and lead to the formation of foam cells [7].

The next stage is fatty streak formation, which initially consists of lipid-laden monocytes and macrophages that are engorged with oxidized LDL (oxLDL) forming foam cells together with T lymphocytes. Subsequently, SMC migration from media to fatty streak increases the number of cells in this area [6]. Thus, in the center of atheromatous plaque, foam cells and extracellular lipid droplets form a core region that is surrounded by a cap of SMCs and a collagen-rich matrix [6, 8].

These events drive to a structural reorganization of ECM and as consequence have a crucial role in altered cellular behavior. In the media portion of vascular wall, the ECM in healthy conditions consists largely of type I and type III fibrillar collagens and elastin, whereas in atherosclerotic lesions it consists mainly of proteoglycans (PGs), such as versican and HA, intermixed with loosely scattered collagen fibrils [9]. PGs represent a special class of glycoproteins that are heavily glycosylated by one or more covalently attached glycosaminoglycan (GAG) chains. These GAG chains are long, linear carbohydrate polymers that are negatively charged under physiological conditions, due to the occurrence of O-sulfation, N-sulfation, and glucuronic/iduronic acid groups. On the other hand, HA, made of the glucuronic acid (GlcUA) and N-acetylglucosamine (GlcNAc) disaccharide repeats, is the unique GAG and is not covalently linked to core proteins and not sulfated or epimerized. HA synthesis is catalyzed by three HA synthases (HAS) located on the plasma membrane [10–12]. HA synthesis, therefore, differs completely from that of the other GAGs, which is a typical process localized in the Golgi. HA synthesis is finely regulated by cytokines, growth factors, and prostaglandins, as well as by the precursor availability (i.e., UDP-GlcNAc and UDP-GlcUA), which modulates HAS gene expression [13, 14]. While collagen inhibits cell growth, PGs and HA stimulate SMC proliferation and migration contributing to vessel wall thickening [15–17]. How ECM can modulate cell growth and motility is only partially known. However, PGs can form highly hydrated ECMs that facilitate migration and diffusion of mitogenic molecules, growth factors, and inflammatory mediators. Moreover, ECM remodeling can release active fragments, known as matrikines, of other signaling compounds bound to PGs and GAGs that easily can reach cell surface receptors [18]. Additionally, HA can interact with several cell surface receptors, including CD44, RHAMM, and TLR2/4 that are largely distributed on different cell types, which can trigger internal cell signaling and modulate differentiation, proliferation, migration, and development [19, 20].

In addition to this complex picture, low molecular mass HA can be produced during inflammation due to oxidative stresses, enzymatic degradation by hyaluronidases, and/or aberrations during the HA synthetic process [17]. Interestingly, it was shown that HA synthesis can be induced in ECs by the action of IL-15 in an inflammation model [21]. Although high molecular mass HA exerts antiproliferative, anti-inflammatory, and protective properties on cells, low molecular mass HA has the surprising ability to activate Toll-like receptor (TLR) 2/4 signaling leading to enhancement cell replication, MMP secretion, and invasive properties [11, 20, 22]. In inflammatory responses, the important role of HA receptors in transition of monocytes to macrophages has been reported [11, 23–26].

This review will describe the known strategies that vascular cells adopt to regulate HA synthesis, which could identify future targets for pharmacological treatments in vascular pathologies.

## 2. HA Regulation in ECs

ECs are the most inner lining in blood vessels that control transport of nutrients and other substances from the bloodstream into the tissues. Moreover, the endothelium regulates vascular SMC tone, blood clotting, and angiogenesis. ECs also have a critical role in the inflammation mediating the adhesion and the extravasation of immune cells. Typical endothelial proteins like ICAM-1, P-selectin, and VCAM-1 are key molecules in adhesiveness of circulating cells.

Glycocalyx, a complex network of membrane-bound PGs, glycoproteins, and HA that luminally covers the endothelium, is the real interface between cells and bloodstream. Many cellular properties depend on the glycocalyx composition and integrity [27]. HA in the glycocalyx has a protective role against platelet adhesion and immune cell-EC interactions and therefore fulfills an antithrombotic and anti-inflammatory role [28–30]. As a proof of concept Nagy et al. [31] demonstrated damage of the glycocalyx after treatment with 4-methylumbelliferone (a well-known HA synthesis inhibitor [32]), EC dysfunction, increased macrophage driven plaque inflammation, and increased atherosclerosis.

The basal membrane produced by ECs in healthy vessels is mainly composed by collagen IV and laminin. In the early stage of the progression of atherosclerosis, which means before the monocytes are recruited, the inflammatory condition changes the basal membrane into a transitional matrix, which contains fibronectin and fibrinogen [33]. Such a switch alters EC response to flow and shear stress and induces NF-*κ*B activating integrins. As a consequence, the new integrin binding to the inflammatory ECM changes gene expression and enhances endothelial permeability [34].

**Figure 1:**
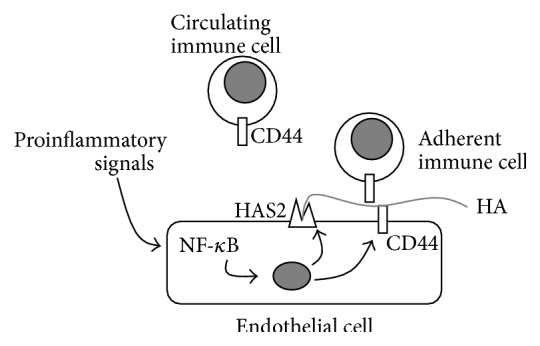
Schematic representation of the regulation of HA synthesis in ECs and the effects on immune cell-EC adhesion. Through their receptors, proinflammatory signals (i.e., cytokines) trigger NF-*κ*B pathway that regulates both HAS2 and CD44 (and other adhesive molecules such as ICAM-1, E-selectin, VCAM-1, and MHC class I genes) [38]. HAS2 synthesizes high molecular weight HA that interacts with CD44 present on both ECs and immune cells (i.e., leukocytes) in the “sandwich model,” which drives immune cells to adhere to ECs contributing to inflammation. Gray circle represents the nucleus.

HA synthesis has been found to be inducible in EC cultures, and its synthesis is triggered by proinflammatory mediators such as IL-1*β*, IL15, TNF-*α*, and lipopolysaccharide [35]. The increased secretion of HA during inflammation is due to the overexpression of the isoenzyme HAS2 [21, 36]. The cellular pathway that leads to HAS2 expression involves a NF-*κ*B pathway. Increased HA, together with an augment of the typical adhesion molecules, is responsible for the increased binding of immune cells to ECs and is critical for regulating tissue inflammation. Interestingly, CD44 is also overexpressed in ECs treated with proinflammatory mediators, suggesting a particular model of EC/immune cell interaction. In this model, newly synthesized HA would be kept above the endothelium by CD44, and leukocytes would be bound to HA through their own CD44 [37]. Thus, this model highlights the importance of HA synthesis and confirms the critical role of HAS2 in triggering HA synthesis after proinflammatory signal treatments.  Figure 1 summarizes the concepts expressed in this paragraph. Together with the alteration of the basal membrane, HA deposition seems therefore to be the earliest signal of the onset of a vascular disease.

## 3. HA Synthesis Regulation in Vascular Smooth Muscle Cells

Vessel wall ECM is mainly synthesized by SMCs in the media. Different from EC, vascular SMC (VSMC) cultures produce large amounts of HA that can be easily found in the conditioned medium as well as in the VSMC pericellular space. High expression of HAS2 and HAS3 is necessary for HA synthesis in VSMCs as well as the presence of the enzymes for production of substrates via the UDP-glucose dehydrogenase and hexosamine biosynthetic pathway (HBP) [36, 39–41]. From a metabolic point of view, the entering of nutrients in anabolic pathways is a very finely tuned process as all the cellular energetic requirements should be satisfied before biosynthesis of HA is allowed [19]. As the synthesis of HA sugar nucleotide precursors is a high energetic demanding process, we found that adenosine monophosphate activated protein kinase (AMPK) deeply affects HA synthesis [38].

**Figure 2:**
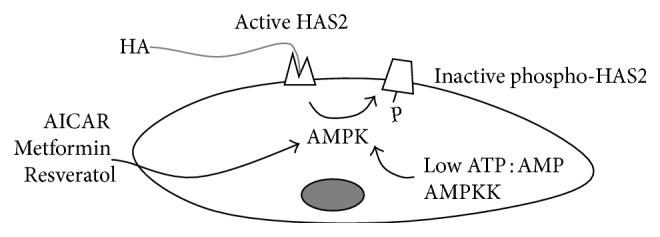
Schematic representation of the regulation of HA synthesis by AMPK in SMCs. Through the action of compounds such as AICAR, metformin, and resveratrol or by sensing ATP : AMP ratio or by the action of AMPK upstream kinases (AMPKK), AMPK phosphorylates HAS2 threonine 110 residue inhibiting HAS2 activity and reducing the HA production.

AMPK has a pivotal role in regulating energy homeostasis in eukaryotic cells. In response to a decrease in cellular ATP levels, AMPK reduces the rate of anabolic pathways (ATP-utilizing) and increases the rate of catabolic pathways (ATP-producing) [42]. Through the capacity to detect the ATP : AMP ratio, AMPK acts as a metabolic master switch and phosphorylates key target proteins that control flux through metabolic pathways in order to maintain energy homeostasis. HAS2 is a substrate of AMPK, which phosphorylates threonine 110 to inhibit HAS2 enzymatic activity [38]. Interestingly, AMPK does not alter the synthesis of other GAGs secreted by VSMCs, highlighting the specificity of AMPK action on HA synthesis. This aspect could explain the vasoprotective effect of AMPK activation, obtained also by the antidiabetic drug metformin, to reduce neointima formation in animal models for atherosclerosis [43] (Figure 2).

**Figure 3:**
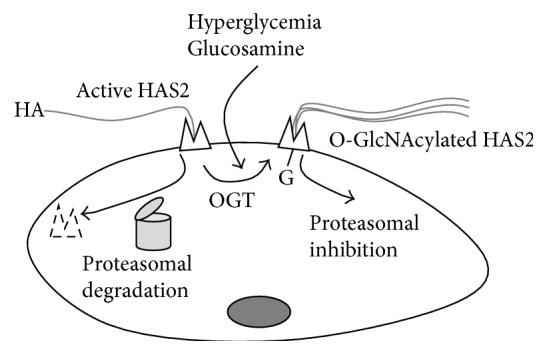
Schematic representation of the regulation of HA synthesis by OGT in SMCs. In normal conditions HAS2 in plasma membrane is active but can be rapidly degraded in a 26 S proteasome dependent manner. In hyperglycemic condition or after glucosamine treatments, OGT catalyzes the O-GlcNAcylation of HAS2 serine 221 residue, which greatly stabilizes HAS2 favoring HA production.

Glucose is a major cell substrate, and its utilization is finely regulated allosterically as well as hormonally. HBP represents an alternative glucose pathway in the cells which leads to the formation of the crucial sugar nucleotide UDP-GlcNAc [44]. Besides being a substrate for HA and other glycoconjugate biosyntheses, UDP-GlcNAc is the substrate of O-GlcNAc transferase (OGT), the critical enzyme for protein O-GlcNAcylation [45], which is the reversible modification of nuclear or cytosolic proteins by attachment of a single *β*-GlcNAc moiety by O-linkage to specific serine/threonine residues. O-GlcNAcylation increased typically in hyperglycemic conditions, due to the entering of the excess of glucose in the HBP, and is involved in several diabetic complications [46–48]. As angiopathies are one of the main complications of diabetes and HA is altered in human diabetic patients [49] as well as in animal models of this pathology [50], it is not surprising that HA synthesis is induced by HAS2 O-GlcNAcylation (Figure 3) [39].

O-GlcNAcylation of HAS2 occurs on serine 221 in the cytoplasmic loop, which stabilizes the enzyme in plasma and cytosolic membranes. This allows the enzyme to have a half-life of more than 5 hours before proteasomal degradation (although in other cell types HAS2 is ubiquitinated [51]). In contrast, HAS2 without this modification only has a half-life of 17 minutes [39].

**Figure 4:**
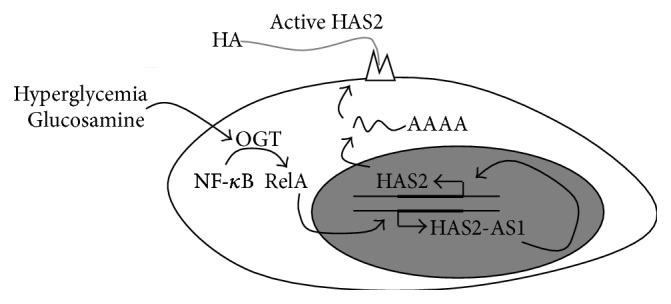
Schematic representation of the regulation of HAS2 expression by OGT in SMCs. In normal conditions, basal HAS2 and HAS2-AS1 expression are allowed. After the induction of O-GlcNAcylation (due to hyperglycemia or after glucosamine treatments), the NF-*κ*B subunit RelA can be modified with O-GlcNAc by OGT. In the cell nucleus, glycosylated RelA can activate HAS2-AS1 transcription, which, in turn, changes chromatin structure around the HAS2 promoter (probably altering chromatin signature) favoring HAS2 expression leading to HA accumulation.

O-GlcNAcylation is also able to modulate gene expression, and in VSMCs this glycosylation leads to an increment of HAS2 mRNA [52]. The molecular mechanism of such regulation is complex and does not involve the typical O-GlcNAcylated transcription factors that are known to regulate HA synthesis like YY1 and SP1, which are able to interact with the HAS2 promoter [13]. By using ChIP analyses from mice with high O-GlcNAcylation levels, we found a significant signal in correspondence with the natural antisense transcript for HAS2 (HAS2-AS1), which is a particular long noncoding RNA transcribed using the opposite strand of HAS2 locus on chromosome 8 [53]. HAS2 and HAS2-AS1 RNA molecules share about 200 base pairs and can form a RNA:RNA duplex. In previous papers antisense transcripts have been described to stabilize their sense mRNA [54, 55], but this mechanism did not work with VSMCs. In contrast, we found that HAS2-AS1 transcription is initiated by O-GlcNAcylation of RelA (a component of NF-*κ*B complex) [56], and HAS2-AS1 was necessary to change chromatin structure around the HAS2 promoter to allow more efficient binding of RNA polymerase 2, thereby enhancing HAS2 gene expression (Figure 4) [52]. In vivo analyses confirmed the crucial role of HAS2-AS1 in the regulation of HAS2 in humans and in animal models for vascular pathologies [52]. Although investigations are still in progress, our hypothesis is that HAS2-AS1 could alter the epigenetic signature of the HAS2 promoter by switching it into a more active chromatin. The more recent theories about the long noncoding RNA functions highlight their role in recruiting enzymes able to modify histones and DNA in particular loci of the genome [57–60].

## 4. LDL and HA Metabolism

Despite the great challenge to discover specific markers for the onset of atherosclerosis, at the moment the unique factors routinely evaluated in the clinical practice are the quantification of circulating LDL-cholesterol and the ratio LDL/HDL. The presence within the intima of cholesterol-rich macromolecules induces the formation of foam cells, originating from both macrophages and SMCs [6, 61] encircled by altered ECM. As recently reported, upon activation with the endotoxin LPS, macrophages undergo activation to the M1 phenotype [62] with a concomitant rapid increase in Has1 mRNA and inhibition of hyaluronidases 1 and 2, the major HA degrading enzymes. Nevertheless, HA deposition could only be detected after inhibiting lysosomal and endosomal activities with chloroquine, indicating a rapid turnover and degradation of HA from the ECM [63]. These results are in line with the increasing evidence of the pivotal role of SMCs as ECM modulators. When SMCs resident in the tunica intima and media [64] get in contact with chemically modified LDL, they produce the inflammatory ECM of the plaques. Given the numerous oxidative events associated with the development of an inflammatory atherosclerotic plaque, LDLs, which are accumulated early in the intima primarily from enhanced retention by the GAGs, are converted into oxLDL or into aggregate LDL (aggLDL). Oxidation is the most representative modification, and several enzymatic and nonenzymatic oxidant candidates have been described to be involved [65]. Oxidation may affect both lipid and protein moieties of the particles, and as a result, a series of biologically active species including peroxides, aldehydes, and oxysterols may be produced [66, 67]. The two different modifications of LDL exert different roles in VSMCs. Only oxLDL induced the neosynthesis and deposition of HA, while aggLDL engulfed the cells with lipids without affecting the ECM.

**Figure 5:**
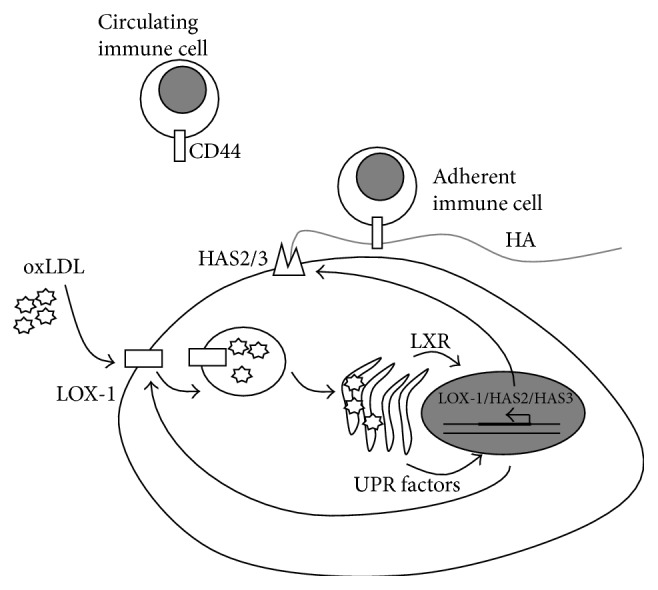
Schematic representation of HA metabolism in SMCs loaded with oxidized LDL (oxLDL). Nontoxic concentrations of oxLDL are driven inside the SMCs by the upregulation of the scavenger receptor LOX-1. Accumulation of oxLDL leads to ER stress with overexpression of the UPR factors CHOP and GRP78 as well as activation of the LXR sterol sensor system. One or both systems induce the overexpression in the nucleus of several genes: LOX-1, HAS2, and HAS3. The HASs are active on the plasma membrane where they synthesize HA that interacts with CD44 present on immune cells, driving their adhesion, which contributes to the inflammatory status of the atherosclerotic lesion.

Several scavenger receptors have been reported to be able to internalize oxLDL (e.g., SR-PSOX, SR-AI, CD36, LOX-1, and LRP1). However, even though some of them are present on the SMC membrane, only LOX-1 was upregulated after oxLDL exposure [68, 69]. The oxLDL entrance via LOX-1 has various effects inside the cells: upregulation of LOX-1, increasing the load of lipids and ROS in ER leading to ER stress, and overexpression of HAS2 and HAS3 with concomitant deposition of HA in the ECM (Figure 5), all of which occur during atherosclerosis progression. The increase in HA in the SMC ECM promotes macrophage adhesion and activation as well as their higher migration ability.

LOX-1 is a well-known receptor expressed by vascular ECs, where LOX-1 mediates oxLDL-induced EC apoptosis via an elevation of intracellular NADPH oxidase, which in turn activates the ER stress pathway [70]. However, remarkably, there is no information given for HA metabolism.

The ER stress rescue (also named UPR, unfolded protein response) mechanism is induced in both SMCs and ECs after oxLDL loading, including the expression of several proteins, including CHOP and GRP78, that can modify the activity of transcription factors in the cell nucleus [71]. This intriguing mechanism has been taken into consideration in the investigation of several ECM molecules, including HA [72, 73]. Given the role of LDL in cholesterol metabolism, a key factor in this context is the LXR/RXR sterol sensor system [74]. Liver X receptors (LXR) of the nuclear receptor superfamily are factors that regulate the transcription of several genes involved in cholesterol metabolism. It is noteworthy that the use of cholesterol depleted ox-LDL fails to stimulate HAS2 overexpression and HA synthesis. Interestingly HAS3 was upregulated by these macromolecules; moreover, cholesterol and oxysterols control the expression of HAS3 while only oxysterols can regulate HAS2 transcript levels [69]. It seems therefore evident that a different control of the two enzymes HAS2 and HAS3, regulated by oxysterols/LXR/RXR and/or UPR factors, can reflect their different role in cell behavior.

## 5. Conclusions

Vessel wall ECM components have a great impact on cell behaviour and, therefore, on vascular pathologies. The critical effect of HA was demonstrated in different ways, and a better knowledge of the mechanisms that control HAS expression could identify future targets for pharmacological treatments of circulatory diseases.

## Abbreviations

ECM: Extracellular matrix
HA: Hyaluronan
PGs: Proteoglycans
GAGs: Glycosaminoglycans
MMP: Matrix metalloprotease
ECs: Endothelial cells
SMCs: Smooth muscle cells
UDP-GlcNAc: Uridine diphosphate-N-acetylglucosamine
HAS: Hyaluronan synthase
AMPK: Adenosine monophosphate activated protein kinase
HBP: Hexosamine biosynthetic pathway
oxLDL: Oxidized low-density lipoproteins.
